# Pathology, Practical Medicine, and Therapeutics

**Published:** 1838-07

**Authors:** 


### Selections from the British Journals.

### PATHOLOGY, PRACTICAL MEDICINE, AND THERAPEUTICS.

*Cases of Acute Inflammation confined to the Epiglottis.* By H. Marsh, M.D. M.R.I.A., one of the Physicians to Stevens' Hospital.

[This is a very valuable practical communication, such as might be expected from the experienced and skilful physician who is the author of it. We can only find room for Dr. Marsh's own cases, and a few of the remarks appended to them.]

Case I. Mrs. T—, between fifty and sixty years of age, robust, and of plethoric habit and florid complexion, had been confined for some days to her bed by a feverish cold. The symptoms having rather abruptly assumed a more serious aspect, I was requested to visit her, having been informed by the medical gentleman in attendance that her case was an obscure one, and that he conceived her disease to be a nervous fever. I found her flushed, agitated, and complaining principally of being restless and sleepless: the moment her eyelids closed, she started suddenly, gasped for breath, and looked like one about to be convulsed. She sat up erect in bed, and would not for a moment venture to lie down. A continual accumulation of tenacious mucus obstructed the fauces, prevented sleep, and produced frequent and urgent paroxysms of dyspnœa; her skin was hot and dry; her pulse 120, contracted and resisting; and, to the enquiry whether she was distressed by thirst, she replied she was afraid to drink, the attempt to swallow was productive of so much pain and suffering. On handing her a glass of water, she seemed to dread to put it to her lips. On making an effort to swallow, a struggle so painful and convulsive, with protrusion of the eyes, ensued, that it gave rise in my mind to the suspicion that she had been bitten by a rabid animal. In attempting to swallow, the fluid was forcibly and convulsively rejected. On examining the interior of the mouth and fauces, I could discover no appearance indicative of inflammation or disease of those parts: the tonsils, uvula, and soft palate, presented a perfectly natural appearance; but the forcible depression of the tongue produced so much pain, and caused such convulsive movement of the muscles, that I was unable to obtain a satisfactory view of the parts which lie more posteriorly. The tone of her voice was scarcely, if at all, altered; the respiration, in the intervals between the paroxysms, was unimpeded, and it was not at any time in the least degree stridulous.

This case fell under my observation early in my professional life, and I felt very much at a loss to account for the symptoms: it occurred to me at the moment to pass my finger along the tongue, and endeavour to ascertain by the touch the cause of the symptoms. On passing the finger over the tongue, as far towards its root as I could reach, I felt a tense, rounded, prominent body, which, upon close examination, was evidently the epiglottis, considerably enlarged and erect. This at once revealed the seat of the disease, and the source of all the distressing symptoms. I had not previously witnessed an instance of this disease, nor had I, in the course of my reading, met with a description of it. My opinion was at once formed: it was now quite certain that I had to do with a case of acute inflammation of the epiglottis, accompanied with high inflammatory fever. The treatment was obvious: the patient was instantly and largely bled from the arm, and leeches numerously and successively applied, so as to maintain for some time a continued flow of blood; the thick tenacious mucus was removed from the fauces from time to time, by means of a sponge attached to a piece of whalebone. After a few hours the symptoms were considerably abated, and some rest was obtained; bleedings from the arm were repeated a second and a third time, and each repetition of the bleeding was followed by a still more evident abatement of the symptoms: she was now able, though with considerable pain and difficulty, to swallow. Calomel and Dover's powder were given in repeated doses; and, the symptoms having continued for some hours stationary, it was determined to introduce mercury by friction, so as to influence the system as speedily as possible. Half a drachm of mercurial ointment was rubbed in every fourth hour: moderate ptyalism was established; the remaining symptoms rapidly subsided, and after the lapse of a few days scarcely a trace of the disease remained. This lady has since enjoyed perfect health, no disposition to a return of the disease having manifested itself.

Case ii. The next very remarkable instance of this affection which fell under my observation occurred in an individual whose constitution and temperament were altogether the reverse of those which characterized the case just related. The disease manifested itself in a young person not twenty years of age, of delicate frame and feeble constitution, and arose in the following manner:—In consequence of exposure to cold, this young lady was affected with slight rigors; fever ensued, accompanied with some tumefaction and redness of the tonsils and uvula. The fever and inflammation yielded to the quiet of bed and to diaphoretic medicines. On the third day she was convalescent. On the evening of the third day, contrary to the injunctions she had received, she got up and exerted herself: on returning to bed, she felt herself chilly and uncomfortable, and spent a feverish and restless night. Next morning she experienced some pain and difficulty in deglutition, and, these symptoms continuing rapidly to increase during the day, I received an urgent message to see her. On visiting her in the evening, I found her in a state of great suffering and distress: she was incessantly harassed with a profusion of tenacious transparent matter, which was generated about the fauces, and every effort to rid herself of this was attended with very great distress; the act of protruding the tongue was extremely painful to her; she feared to lie down; there were frequent paroxysms of dyspnœa; she was agitated and apprehensive to an extraordinary degree; her friends around her were in a state of the greatest alarm; every effort to swallow was accompanied with violent pain, the liquid was rejected with force through the nostrils, and her distress and agitation were much increased. On examining the fauces there was scarcely a remaining blush, and the tumefaction which had existed on the first attack had entirely subsided: it was impossible sufficiently to depress the tongue to obtain a view more posteriorly, but, on introducing the finger, the epiglottis was found greatly swollen, tense, and smooth, and pressure with the finger produced severe pain; there was no external swelling; she experienced some uneasiness on pressure upwards at the highest part of the neck; the face was darkly flushed, anxiety and suffering were strongly depicted on the countenance, and the pulse was incalculably frequent, small, and feeble; the skin was hot, and covered with a clammy perspiration; the voice was natural; there was no cough, but the efforts to clear away the phlegm from the throat were almost unceasing; the respiratory and percutatory sounds were perfectly natural. Leeches were applied externally, as near as possible to the seat of the disease, and fomentations with the decoction of poppies, after the removal of the leeches, perseveringly employed. I visited her again very late at night, accompanied by Mr. Cusack, whose cooperation I was requested to obtain. There was no abatement, rather an increase, of all the urgent symptoms. Mr. Cusack, by sewing a dossil of lint to the end of the finger of a glove, and putting it firmly on his forefinger, was enabled to remove a large quantity of the adhesive mucus from the fauces, and thus procure considerable temporary ease; a short interval of sleep, in the erect position, was thus obtained: leeches were again applied, and fomentations repeated. No medicine could be given by the mouth: purgative injections were administered, and acted well. At our next visit, though no positive abatement of the symptoms could be said to have taken place, yet the patient on the whole seemed somewhat less agitated and distressed: the fauces were again cleansed in the same manner as before. It was now determined in consultation to apply, by means of the dossil of lint attached to the finger of a glove, a solution of the nitrate of silver (ten grains to an ounce of distilled water) to the inflamed epiglottis. At first some pain, afterwards marked relief, ensued from this application, which was repeated at every subsequent visit, the strength of the solution being gradually increased; and it appeared to me that its employment was always attended with benefit. We also determined to commence at once mercurial treatment, the urgency of the symptoms demanding its immediate application. A drachm of mercurial ointment was accordingly ordered to be rubbed in on the inside of the thighs, every fourth hour; and, in addition, to excite as speedily as possible mercurial action in the system, the surface of the body under the bedclothes was exposed to the continual contact of volatilized mercury: the bedclothes being retained in an elevated position, this was readily accomplished. The vapour arising from the hydrargyrum cum creta, heated, was generated abundantly, and retained between the bed-coverings; and, by perseverance in this, the entire surface of the patient's body was kept constantly involved in a mercurial atmosphere. On the third day from the commencement of this treatment, moderate but decided ptyalism was established; the occurrence of which was coincident with a marked and rapid abatement of every distressing symptom. Previously to the constitution having been placed under the influence of mercury, a mitigation of the symptoms had been effected. On the third day the patient was able, though with great pain, to swallow small quantities of fluids; and short but refreshing intervals of sleep were procured. It was not, however, until the end of the fifth day, when the decided action of mercury on the system became apparent, that a complete and permanent subsidence of the symptoms was manifested. After this period deglutition became easy, the paroxysms of dyspnœa no longer recurred, and the sleep was tranquil and refreshing. Towards the end of the third day the epiglottis felt rough to the touch, but still swollen and large: afterwards the feel was that of a body that was wrinkled and puckered, but not much swollen. After the lapse of many days, I examined again the epiglottis, and the touch could detect no abnormal condition of this organ. Convalescence was slow and tedious.

Case iii. The next case was that of a man aged forty, who, after exposure to cold, was affected with much pain and difficulty of swallowing, the liquids regurgitating through the nose in every effort. On looking into the mouth, the tonsils, soft palate, and uvula presented a natural appearance; but at the base of the tongue was seen a round, red, prominent substance, like a small ripe cherry. The attendant fever was considerable. For three days he was unable to swallow anything: he was relieved by frequent relays of leeches; warm baths and anodyne enemata having been used without apparent benefit. The constant oozing of blood from the leech-bites appeared to be the only effectual part of the treatment. On the third day the inflammation began gradually to subside, and the patient ultimately but slowly recovered.

*Remarks.* The anatomical characters and relations of the epiglottis fully explain the nature and course of the symptoms detailed. Being studded with glands at its root, the inflammatory irritation stimulates them to a greatly increased secretion of mucus: this adhesive mucus, in constant efforts to detach it, greatly harasses the patient, produces paroxysms of dyspnœa, and prevents sleep. The connexion of the epiglottis with the root of the tongue accounts for the pain felt when the tongue is moved or protruded, and its situation and the relation which it bears to the muscles of deglutition fully account for its essential symptoms,—pain, spasm, and difficulty in the act of swallowing. The loose attachment of the mucous membrane to its anterior surface will explain satisfactorily the great extent of the tumefaction, and also that the inflammatory distention should be situated on the lingual rather than on the laryngeal aspect of the organ, as well as for the puckered or wrinkled feel of the mucous membrane on the absorption of the effused fluid. . . . . . .

In the cases detailed, the inflammatory action was limited in extent, not extending probably beyond the anterior surface of the epiglottis. The circumscription of the inflammation was marked by positive as well as negative signs. The inspection of the fauces proved that it did not exist in the parts anterior to the epiglottis; the absence of dyspnœa, except in paroxysms; of stridulous breathing; the natural tones of the voice when the mucous matters were detached; the exemption from cough; the result of stethoscopic examination: all these negative signs prove that neither the glottis, larynx, nor bronchial mucous membrane shared in the inflammatory action. These cases are therefore examples of unmixed, circumscribed inflammation of the epiglottis.

*Treatment.* Slight cases of this disease are not unfrequent, but require little or no treatment. In cases of medium severity, the symptoms are not very urgent, and will, with mild antiphlogistic treatment, subside; but, in the more acute and intense examples of this disease, the urgency of the symptoms and the sufferings of the patient demand the most prompt and vigorous treatment. From the cases I have observed, and from those recorded, I am disposed to gather that the disease has a tendency to abate either on the third, fifth, or seventh days; but, if not combated by energetic remedies, it may end in suppuration, as it did in one recorded case; or it may extend downwards, involve the glottis, and thus produce a still more dangerous disease, œdema of the glottis; or, by causing repeated paroxysms of dyspnœa, it may give rise to pulmonary infiltration; or it may but partially subside, and leave behind a thickened, indurated, and permanently diseased condition of the epiglottis. To allay the urgent symptoms, and prevent these consequences, active treatment, regulated of course by the patient's constitution, is imperatively required. In one of the cases I have recorded, frequent and large bleedings, general and topical, with the active administration of mercury, were necessary to reduce both the concomitant fever and the local inflammation. In another, the delicate constitution, the feeble pulse, the state of the skin, induced me to restrict bleeding to the reiterated application of leeches as near as possible to the seat of the disease. To subdue inflammatory action, as well as to prevent lingering chronic disease, mercury is invaluable. Fortunately it can be applied as effectually, and I think as rapidly, by the skin, as when administered internally. In cases such as those now detailed, where the ability to swallow is lost, its external application, both by friction and by vapour, is of immense value; and it seems to me that, in the more severe cases of the disease, this part of the treatment should not be omitted. I think the application of the nitrate of silver, as suggested and practised by Mr. Cusack, was decidedly useful. Fomentations long continued, though a minor remedy, are not without their value. Blistering I did not think necessary, nor would I apply a blister till the inflammatory excitement were markedly reduced.

From the administration of tartar emetic at the period of commencing restoration of the power of deglutition, I abstained, fearful of exciting vomiting; which would, I conceive, be distressing, perhaps dangerous to the patient. Were, however, the fever to continue, and the symptoms not to yield satisfactorily, it might be given, guarded with opium in carefully regulated doses. The influence of this combination of medicine, in diminishing excitement and reducing inflammation, is established incontestibly in various conditions of disease; but the symptoms in the cases recorded yielded so completely to bleeding, mercury, and fomentations, that I did not deem it necessary to resort to other means; and in none of them has any trace of chronic disease been left behind.

*Dublin Journal of Med. Science. March,* 1838.

---

*On Tapping the Head in Hydrocephalus.* By T. J. CONQUEST, M.D.

[THIS is an important paper, and affords ample testimony of the great value of the practice, for the introduction of which into medicine we are all deeply indebted to Dr. Conquest. We give the more important parts of this communication in the author's own words; and must, at the same time, express our approbation of the modest and philosophical tone that pervades it.]

Nearly ten years having elapsed since I was first induced to attempt the cure of chronic hydrocephalus by withdrawing the fluid from the ventricles, the time seems to have arrived when the profession has a claim on me for some account of the results of these operations; and, indeed, this has become necessary in consequence of the numerous applications for information on the subject by practitioners, not only in Britain, but in many distant parts of the world. Still, it is a matter involving such important considerations, that, until experience has thrown much more light upon it, I do not feel justified in advancing anything beyond the mere statement of facts, such as the present position of my enquiries warrants, leaving to a future day a more methodical and full investigation of the origin, nature, and progress of this formidable disease, with its appropriate medical and surgical treatment.

The operation consists in passing a small and delicately constructed trocar into one of the lateral ventricles, and drawing off so much fluid as the powers of the constitution will admit of. The most eligible spot at which the trocar can be introduced is in the course of the coronal suture, about midway between the crista galli process of the ethmoid bone and the anterior fontanel, so that the danger of wounding the corpus striatum is avoided on the one hand, and the longitudinal sinus on the other. The instrument usually penetrates about two inches, and in most cases the serum has been colourless, but occasionally tinged with blood. In one instance, and that was in the last child operated on at St. Bartholomew's only a few weeks since, a large and alarming quantity of florid blood escaped; most likely from a branch of the meningeal artery. Sometimes, on withdrawing the trocar, the water will not flow until a probe has been passed along the canula, to remove portions of cerebrum which block it up. After taking away all the fluid that can be removed consistently with safety, the head, which should always be steadily compressed by an assistant during the operation, may be strapped with adhesive plaster, that it may retain its diminished size, and that the fearful consequences of suddenly removing long-continued pressure from the brain may be averted.

I have now tapped in *nineteen* cases, and of these *ten* were living when last heard of. Several of the children, before the operation, were reduced to the most deplorable condition, having frequent convulsions, with loss of sight, emaciation, &c.; but the diminution or disappearance of these symptoms has been very remarkable. In some cases the results have been triumphantly successful; in others, from the reluctance of the parents to have the operation repeated, only temporary relief has been afforded; but none of these children died either during or immediately after the operation; and those which, in the subsequent list, are reported as dead, survived weeks or months after the fluid was withdrawn.

All the operations were performed in the presence of many medical men, and most of them before large bodies of students, at St. Bartholomew's Hospital, and their progress has been watched by gentlemen who felt a deep interest in their termination; and, although exclusive dependence has been placed on the withdrawal of the fluid, without the auxiliary assistance of any pharmaceutical or other medical means, yet I consider much of the success to be attributable to the kind and able superintendence of medical friends.

| No. | Name. | No. of times operated on. | Quantity withdrawn. | Living. | Dead. |
|---|---|---|---|---|---|
| 1 | Catherine Seager .......... | 1 | ℥ xxxij. | 1 | 0 |
| 2 | William Honey ............ | 3 | xxxivss. | 0 | 1 |
| 3 | William Wilmer ........... | 2 | xxiv. | 1 | 0 |
| 4 | John Hall .................. | 5 | xlviijss. | 0 | 1 |
| 5 | Alfred Parman ............ | 4 | xlv. | 0 | 1 |
| 6 | Mary Ragon ............. ... | 3 | xxvj. | 1 | 0 |
| 7 | Charles Discomb ......... | 2 | xx. | 0 | 1 |
| 8 | John Ward ............... ... | 1 | viiij. | 0 | 1 |
| 9 | John Clauditt............... | 2 | xxij. | 0 | 1 |
| 10 | Charles Clarke .. ......... | 2 | xvij. | 0 | 1 |
| 11 | Elizabeth Forster ........ | 5 | lv. | 1 | 0 |
| 12 | Jemima Evans ............. | 1 | vijss. | 0 | 1 |
| 13 | Jane Brocken ................ | 1 | xiij. | 1 | 0 |
| 14 | Eleanor Mahoney ........ | 1 | ix. | 1 | 0 |
| 15 | Francis Chiddy ............ | 4 | xxxiij. | 0 | 1 |
| 16 | Thomas Norman .... ...... | 1 | vj. | 1 | 0 |
| 17 | Anne Armenio .............. | 3 | xxxjss. | 1 | 0 |
| 18 | James Thomson............ | 2 | xiv. | 1 | 0 |
| 19 | John Pratt.................. | 1 | ix. | 1 | 0 |
| 19 | | 44 | 455 | 10 | 9 |

*Lancet. March* 17, 1838.

---

*Case of Empyema.* By T. OGIER WARD, M.D., Physician, Shrewsbury.

[THIS case presents some interesting features, which the excellent pathologist and auscultator who treated it so judiciously, did not fail to remark.]

Sept. 14th, 1837.—Joseph Hadduck, æt. two. In April last (four months ago) he was seized with cough and other symptoms of inflammation of the chest, for which he was treated by a druggist. . . .

Present state:—Great emaciation, but little fever; no night-sweats; pulse 160; tongue clean; appetite good; hacking cough, without expectoration; bowels regular. Sleeps only on left side: left side of chest distended, and dull on percussion, with evident fluctuation; no respiratory murmur over that side, except at the upper part and roots of the lung, where it is bronchial. Heart is felt to the right of the sternum. Respiration clear and puerile over right lung. I ordered mercurial friction to the side, and a mixture containing the iodide of potassium.

18th.—As no improvement had taken place, but the emaciation seemed increased, I requested my friend Mr. Crompton to puncture the chest; which he did, and drew off through a canula above a pint of creamy inodorous healthy pus, without the admission of more than a very minute quantity of air into the chest. During the operation the child coughed frequently, and at each effort the pus was projected in a powerful jet through the canula. After the operation, the left side of the chest was as much smaller as it had previously been larger than the right,—

that is, about three-fourths of an inch; but the heart retained its unnatural position, and the child continued to lie on the left side. No bad symptom followed the operation: on the contrary, there was marked improvement. Nevertheless, the chest filled again, and was again emptied on the 25th. On this occasion the pus was the same in consistence and quantity as before; but it had a greenish tinge, and the external air entered the chest freely, though some of it was withdrawn again by an elastic gum bottle. Percussion of the left side immediately became very clear, but no dashing of fluid was heard upon shaking the patient.

26th.—The child looks much better, and is gaining flesh decidedly; he is lively, and his appetite is keen. The pulse has fallen forty beats since the operation, being now 120 instead of 160. Cough slight; left side much smaller than right, and left shoulder lower, the spine being curved laterally. The heart is still to right of sternum; percussion clear; amphoric sound of dashing of fluid on succussion; respiration only audible at back and roots of lung. . . . The chest again became filled with fluid and air, that gurgled audibly upon moving him even slightly; and, on October 7, he was tapped for the third time, and a similar quantity of pus removed in my absence. . . .

12th.—Fluid increased in quantity, but, from its dash, is evidently thinner than before; cough still less, and sweating has ceased; respiration is clear at back of chest.

17th.—Much better; gains flesh fast. Fluid is less, and, judging from the sound, is of thicker consistence. Heart almost in natural position; respiration more extensive, with a little mucous rattle; no cough nor sweating; pulse 100. From this time the fluid became gradually less, and more dense, till it ceased to be detectible by percussion and succussion. The heart returned to its proper place, the spine became straight, and the ribs expanded: the only symptoms left at the end of October being a little obscurity of respiration, and occasional intermittence of the pulse. The child is now (March 10) in perfect health.

The medical treatment, after the first operation, consisted merely in regulating the bowels by occasional doses of hydr. c. cretâ, and in allaying the cough and arresting the perspirations by a linctus containing quinine and acid. sulph. dil. The skin of the chest was drawn aside at each operation, in order to form a valvular opening. . . .

I need not enlarge upon the utility of fluctuation as a diagnostic (I might almost say a pathognomonic) sign of effusion, although I am not aware of its having as yet been applied to the diagnosis of chest disease. In this case, owing to the thinness and elasticity of the parietes of the chest, the fluctuation of the fluid was very distinct on percussion, and in fact formed a useful guide for the introduction of the trocar; since, for want of knowledge of the exact height attained by the effusion, there was some risk of wounding the lung. Another circumstance that was of great use in determining the situation of the puncture in the second operation, was the fluctuation of the fluid caused by the heart's impulse, that was very evident over the intercostal spaces as high as the effusion existed, but there ceased. This sign I once saw exhibited in a remarkable degree in a case of gangrene of the lung, with destruction of the intercostal muscles, and hydro-pneumo-thorax. As this is the only case in which I have been able to detect the exact level of the fluid by this means, it would be presumptuous to offer more than an opinion that it may be of service in ascertaining the more moderate effusions of acute pleurisy and pericarditis; and I conceive its utility would be restricted to indicating the *presence* only of a fluid, notwithstanding that M. Piorry speaks of a gelatinous elasticity communicated to the finger by percussion over an hydatid cyst. Combined, however, with the next sign that I am going to mention, it affords an accurate notion of the *consistence* of the fluid also. This sign, the sound on succussion, has not, to my knowledge, been noticed by any author, except perhaps by Hippocrates, as indicative of the nature and consistence of the fluid effused into the chest. In the present instance, after the air had been admitted, upon shaking the child, a dash of liquid against the walls, and its fluctuation, were perceptible by the hand, as well as audible to the ear at a distance; and, as the fluid became thicker or thinner, augmented or diminished in quantity, so did the dash become smart or heavy, and the sound acute or dull; so that, after the last operation, finding that, as fluid decreased in the smartness of its impulse, the dulness of its fluctuating sound was increased, I concluded that it was becoming absorbed, and that no further operation would be required. It is difficult to describe these varieties of sound and impulse; but any person may convince himself of the possibility of drawing such distinctions, by shaking oil or treacle, and water or spirit, in separate elastic bottles, when not only the state of plenitude of the vessel will be readily ascertained, as Laennec has already pointed out in his article on Pneumo-thorax, but also the density of the contained fluid: indeed, the difference of sound between water and spirit is very apparent.

*Med. Gazette. March* 31, 1838.

---

*Case of Dropsy of the Pineal Gland.* By S. S. Stanley, Esq., Surgeon, Chelsea.

This case is somewhat curious, from the brief period of the disease and peculiar circumscription of the pathological changes.

Three weeks before her death, the patient, a little girl, æt. four, was affected with some fever and restlessness, and afterwards drooped and moped. Four days before her death, "she ran in a hurried manner towards her mother, to whom she clung convulsively, and then fell down suddenly in a state of insensibility, from which it does not appear she ever for a moment returned to consciousness, further than on one occasion giving her mother a kiss." When seen by Mr. Stanley, the day before her death, she was "in bed, lying quite insensible, with the eyes imperfectly closed, and the mouth slightly open; respiration natural; pulse sixty, small and irregular;" and she remained in this state until she died.

*Dissection.* "There was nothing peculiar seen until the brain was removed, when the pineal gland was found distended with fluid to the size of a large hazelnut. The tunica arachnoidea was separated from the gland without difficulty, leaving it entire: the colour of this body was lighter than natural; the pedunculi not apparently implicated; the sac was of various thickness, being thinnest at its upper part, where the density was about a line; in the inferior part, or the basis, it was about two lines in thickness. The enlarged body pressed in a conspicuous manner against the nates of the corpora quadrigemina, and laterally against the thalami optici. The tubercula quadrigemina, and pons varolii, were in a state of ramollissement, probably arising from the pressure described."

*Lancet. March* 24, 1838.

---

*An Account of two Cases of Bronchial Polypus; with Remarks.* By John North, F.L.S.

This paper is an interesting addition to our knowledge of the affection to which it relates. It professes to contain no fresh facts; but we do not recollect to have seen noted, in other cases of the same kind, the stethoscopic indications. The cases are well detailed, and the remarks show learning and sound practical sense.

*Med. Gazette. May* 19, 1838.

---

*Two Cases of Hydrophobia: remarkable Effect of Morphium administered endermically.* By John Burne, M.D., Physician to the Westminster Hospital.

The only thing remarkable in these two fatal cases was the effect of morphium in the first, that of a youth, aged seventeen. It may be well to observe, that although the symptoms were decidedly hydrophobic, there is no proof that they originated from the bite of a rabid animal. We give the conclusion of the case in Dr. Burne's own words:

"He was now materially changed for the worse, and fast approaching dissolution, of which he was fully conscious. He was frequently eructating and retching, throwing up bile and ropy mucus. He was sweating profusely, and struggling with intense crampy spasms of the upper and lower extremities, calling and hollowing out, as the spasms seized him, for three or four persons to rub him, one to each extremity. The eyes were staring, the face was frequently and suddenly contorted, and the head twisted round to the right: at times the trunk was arched suddenly backwards, (opisthotonos) by violent spasms of the dorsal muscles; and spasms of the diaphragm interrupted the respiration almost to suffocation, so that he could speak only in broken words or sentences. He begged to have his throat rubbed, it was so painful from perpetual, distressing, ineffectual efforts to swallow. Often he desired gruel, which, when presented to him, he dare not put to his lips, but would dip his finger into it, and carry this to the roof of his mouth, afraid to approach the muscles of deglutition. He was giddy, recollected with difficulty, moaned distressingly, and altogether presented a picture of suffering and distress such as I never witnessed. The pulse could be felt, but not counted, owing to its rapidity, as well as to the spasmodic twitchings of the tendons and perpetual movements of the body.

"In this state, Mr. White and I considered what could possibly be done to assuage the poor fellow's sufferings. Medicine could not be given by the mouth, not even put upon the tongue, nor by injection. I suggested the endermic use of morphium: accordingly, about ten grains of the acetate were sprinkled on the blistered surface along the spine, and we waited the effect. Scarcely had one minute elapsed when we observed the stare of the eyes and the dreadful alarm and anxiety of the countenance to diminish, then the violence of the spasm to abate, and the catchings in the respiration and the retching to subside; and to our astonishment this general amelioration progressed, till, in four minutes, the countenance had become placid and the respiration free; the retching had ceased, and the spasms vanished! Advantage of this repose was taken to give him some brandy and gruel, and afterwards some broth; of all of which he drank eagerly by a convulsive effort from his own hand, and said they warmed and comforted him. He then fell quietly back, as one fatigued and sleepy, lay down on the pillow on his right side, with his hand under his head and his knees drawn up in a most natural and comfortable position, free from pain and spasms. In this tranquil state we watched him for a quarter of an hour, and having shut out the light and directed the attendants to withdraw from his bed-side, in the hope that sleep might ensue, we retired; leaving instructions, that should the effect of the morphium pass away and the spasms recur, the morphium should be again administered, so as to keep him under its influence; also that a narrow blister should be applied in the region of the diaphragm, to facilitate the further use of the morphium, if requisite. So wonderful an effect from medicine I never saw. At eleven o'clock at night we visited him again. The influence produced by the morphium had continued, more or less, for three hours, when the spasms and other symptoms having recurred, the acetate of morphium was a second time applied, and a second time the symptoms yielded, though not so entirely as before, nor until nearly half an hour after the application. The influence of this second application had now passed away; the symptoms had returned, though in a mitigated form, especially the cramps of the extremities, which have scarcely troubled him since the first use of the morphium. The powers of life, however, are fast declining, the pulse is barely perceptible, and the face is growing sharp. We, nevertheless, did not think it right to abandon the case without another effort; Mr. White, therefore, raised the cuticle by passing a hot iron over wet silk laid upon the epigastrium, and to this fresh surface more morphium was applied, but in vain: the decline of the powers of life had probably rendered the body no longer susceptible. We left him about one o'clock, sinking, and he died in the course of two hours, having become outrageous and frantic some short time previous to dissolution. Death took place early on the sixth day of general indisposition, and in about forty-four hours after the supervention of the hydrophobic symptoms." *Med. Gazette. April* 14, 1838.

